# Relationship Between Lipid Profiles and Hypertension: A Cross-Sectional Study of 62,957 Chinese Adult Males

**DOI:** 10.3389/fpubh.2022.895499

**Published:** 2022-05-18

**Authors:** Siwei Chen, Wenke Cheng

**Affiliations:** ^1^Department of Cardiovascular Medicine, The Third Hospital of Nanchang, Nanchang, China; ^2^Medical Faculty, University of Leipzig, Leipzig, Germany

**Keywords:** hypertension, lipids, total cholesterol, low-density cholesterol, triglycerides, high-density lipoprotein cholesterol

## Abstract

**Background:**

Patterns of dyslipidemia and incidence of hypertension have been rarely reported in Asian populations with inconsistent findings. To accumulate further evidence in Asian populations, the study aimed to investigate the relationship between lipid profiles and hypertension in Chinese adult males.

**Methods:**

We conducted a cross-sectional study based on the data from the DATADRYAD database. The overall population was divided into hypertensive and non-hypertensive groups based on baseline blood pressure levels. For continuous variables, Mann-Whitney test was performed between two groups, while Kruskal-Wallis and Dunn tests were used among multiple groups. The chi-square test was carried out for dichotomous variables. Spearman's correlation coefficient was employed to assess the association between systolic blood pressure (SBP), diastolic blood pressure (DBP) and lipid profiles, whereas the relationship between lipid profiles and the incidence of hypertension was evaluated using multivariate logistic regression. The Bayesian network (BN) model was adopted to investigate the relationship between clinical characteristics and hypertension, and the importance of related predictor to the incidence of hypertension was obtained to make conditional probability analysis.

**Results:**

Finally, totally 62,957 participants were included in this study. In the lipid profiles, total cholesterol (TC), low-density cholesterol (LDL-c), and non- high-density lipoprotein cholesterol (non-HDL-c) were higher in the hypertensive population (*p* <0.001). In the fully multivariate model, for every 1 mg/dl increase in TC, LDL-c and non-HDL, the risk of hypertension increased by 0.2% [1.002 (1.001–1.003)], 0.1% [1.001 (1.000–1.002)], and 0.1% [1.001 (1.000–1.002)]. Meanwhile, HDL-c became positively associated with the incidence of hypertension (*p* for trend < 0.001) after adjusting for the body mass index (BMI), and 1 mg/dl increment in HDL-c increased the risk of hypertension by 0.2% [1.002 (1.000–1.002)] after fully adjusting for multiple variables. Furthermore, the BN showed that the importance of age, BMI, fasting plasma glucose (FPG), and TC to the effect of hypertension is 43.3, 27.2, 11.8, and 5.1%, respectively.

**Conclusion:**

Elevated TC, LDL-c, and non-HDL-c were related to incidence of hypertension in Chinese adult males, whereas triglycerides (TG) was not significantly associated. The relationship between HDL-c and hypertension incidence shifted from no association to a positive correlation after adjusting for the BMI. Moreover, the BN model displayed that age, the BMI, FPG, and TC were strongly associated with hypertension incidence.

## Introduction

Hypertension is not only an important worldwide public health challenge, but also a major contributor to the global burden of disease and death. The global prevalence of hypertension is on the rise, 26.4% in 2000 and is expected to reach 29.2% by 2025 (1). In China, the prevalence is reported to be as high as 44.7% among adults aged 35–75 years (2). Hypertension is a multifactorial disease, with various influencing factors interacting with each other (3). Therefore, identification of risk factors for hypertension and effective early prevention are essential to lower the public health burden.

It is well-known that hypertension and dyslipidemia are the two major risk factors accounting for cardiovascular disease (4). Clinically, hypertension and dyslipidemia often coexist, which may be associated with the fact that they share the same pathophysiological mechanisms, such as endothelial dysfunction (5) and obesity (6). Furthermore, it has been shown that there is a synergistic effect between hypertension and dyslipidemia, indicating that the risk of death and cardiovascular events is significantly higher in patients with both disorders than the combined risk of hypertension and dyslipidemia alone (7). Hence, the association between dyslipidemia and hypertension, further studies are required. Previously, it is shown that dyslipidemia is closely related to the development of hypertension, but most of them have focused on Europe and the Americas (8–12), with relatively few and still inconsistent findings in Asia (13, 14). Therefore, to further accumulate evidence in Asian populations, this study tends to investigate the differences in lipid profiles between hypertensive and non-hypertensive populations in Chinese adult men.

## Methods

### Study Design and Data Extraction

A cross-sectional study was involved, and the original data were downloaded for free from the DATADRYAD database (www.datadryad.org) and provided by Chen et al. (15). The original study aimed to assess the association of the body mass index (BMI) and age with incident diabetes in Chinese adults. From 2010 to 2016, 211,833 Chinese adults were recruited at 32 health centers in 11 Chinese cities (Shanghai, Beijing, Nanjing, Suzhou, Shenzhen, Changzhou, Chengdu, Guangzhou, Hefei, Wuhan, and Nantong). All participants completed a detailed questionnaire assessing demographics, lifestyle, and family history of chronic disease at their initial visit to the health center. Clinical measurements, including weight, height, and blood pressure, were performed by trained staff, when biochemical parameters cover cholesterol (TC), high-density lipoprotein cholesterol (HDL-c), low-density lipoprotein cholesterol (LDL-c), serum creatinine (Scr), blood urea nitrogen (BUN), alanine aminotransferase (ALT), and aspartate aminotransferase (AST). In addition, the BMI was calculated by dividing body weight by the square of height. All data were collected under standardized conditions and according to a uniform procedure.

According to the CC0 1.0 Universal (CC0 1.0) Public Domain Dedication license, as Chen et al. waived all copyright and related ownership rights of the original data, these data could be used for secondary analysis without infringing the rights of the authors. The original study was approved by the Rich Healthcare Group Review Board, while the baseline information was retrieved retrospectively, and the Rich Healthcare Group Review Board has abandoned the requirement for informed consent (16). This study complies with the *Declaration of Helsinki*.

### Study Population

**Figure 1:**
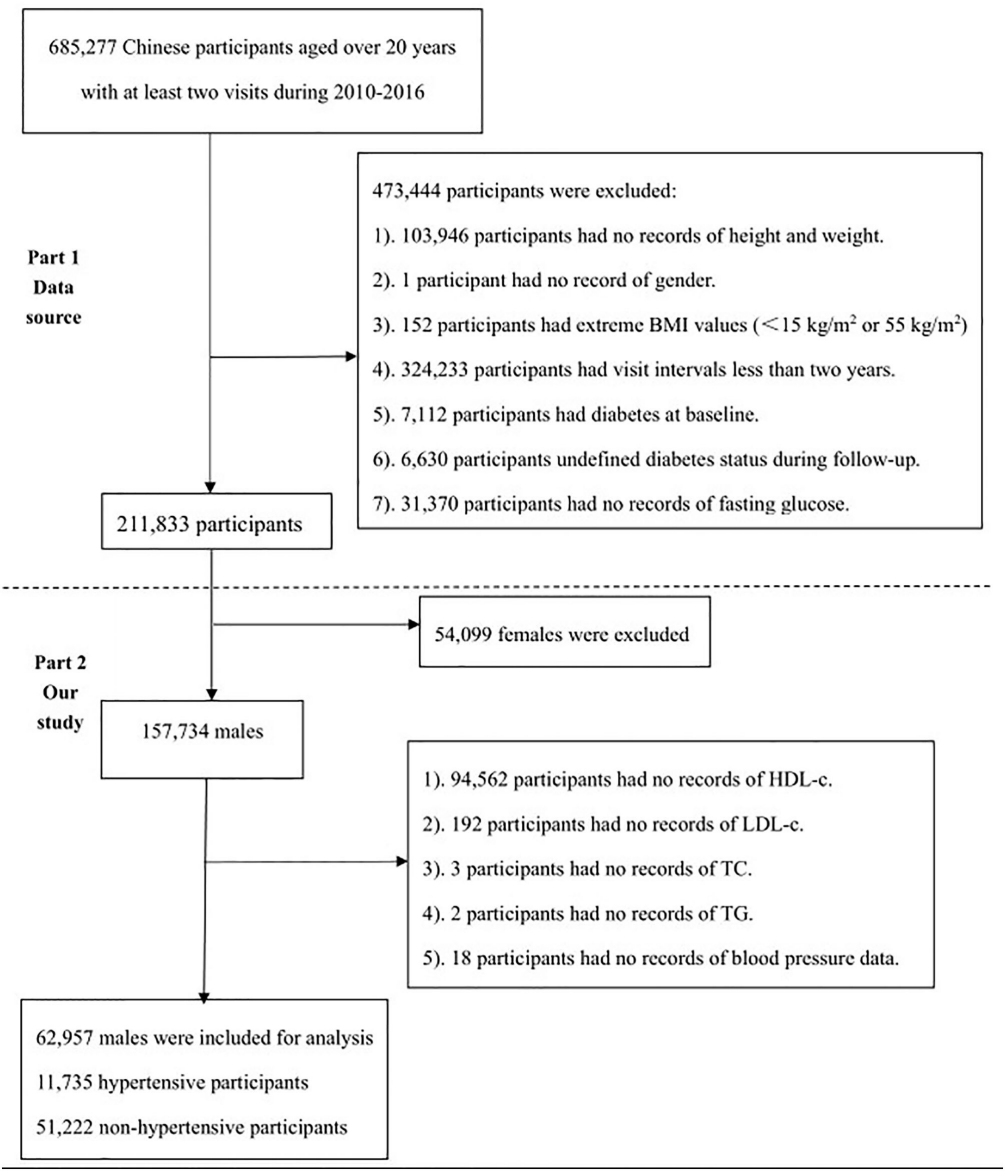
The flowchart of study participants.

From 2010 to 2016, totally 685,277 Chinese adults were initially recruited at 32 health centers in 11 Chinese cities. As shown in Figure 1, the selection of the study population consisted of two parts. The first part was consistent with the original study, with 211,833 participants ultimately analyzed. The reasons for exclusion were presented as follows: (1) Participants had no record of height and weight (*n* = 103,946); (2) Participants had no record of gender (*n* = 1). (3) Participants had an extreme BMI (<15 or 55 kg/m^2^) (*n* = 152). (4) Participants featured a visit interval <2 years (*n* = 324,233). (5) Participants suffered from diabetes at baseline (*n* = 7,112). (6) Participants were at undefined diabetic status during the follow-up (*n* = 6,630). (7) Participants had no record of fasting glucose (*n* = 31,370). However, the second part shows the selection process. Finally, 62,957 participants, including 11,735 hypertensive and 51,222 no-hypertensive participants were involved. Specific reasons for exclusion were shown below: (1) Participants were female (*n* = 54,099). (2) Participants had no records of HDL-c (*n* = 94,562). (3) Participants had no records of LDL-c (*n* = 192). (4) Participants had no records of TC (*n* = 3). (5) Participants had no records of TG (*n* = 2). (6) No blood pressure data were recorded (*n* = 18).

### Exposure of Interest and Outcomes

The exposure of interest was lipid profiles, including TC, TG, HDL-c, LDL-c, and non-HDL-c. non-HDL-c was calculated by subtracting HDL-c from plasma TC levels. With reference to the *Chinese Guidelines for the Prevention and Treatment of Hypertension* (2018 version) (17), participants were classified into hypertensive and non-hypertensive groups based on baseline blood pressure levels, when hypertension was defined as systolic blood pressure (SBP) ≥140 mmHg or diastolic blood pressure (DBP) ≥90 mmHg, and it was further subdivided into mild hypertension (Class I; SBP 140-159 mmHg or DBP 90-99 mmHg), moderate hypertension (Class II; SBP 160-179 mmHg or DPB 100-109 mmHg), and severe hypertension (grade III; SBP ≥180 or DBP ≥110 mmHg), while the non-hypertensive referred to SBP <140 and DBP <90 mmHg.

The primary outcome was to assess the relationship between lipid profiles and the incidence of hypertension, and the secondary outcome was to construct a Bayesian Network (BN) model to evaluate the potential relationship and importance between baseline characteristics and the incidence of hypertension, while using a conditional probability table (CPT) to reflect the probability between the variables and the incidence of hypertension.

### Statistical Analyses

Due to the skewed distribution of the data, continuous data were expressed as the median and interquartile range (IQR), while categorical data were indicated as the number (percentage). The Man-Whitney test was performed concerning differences between two groups for continuous variables; while the Chi-square test was conducted for differences between two groups for categorical variables. Spearman's correlation coefficient was used to assess the association between SBP, DBP and lipid profiles. Males were divided into four groups based on blood pressure levels: normal, grade I, grade II, and grade III. Then, multiple comparisons of lipid levels were performed by Kruskal-Wallis one-way analysis of variance (ANOVA) and the Dunn test, while subgroup analysis was made according to age (<60, ≥60 years), the BMI (<23, ≥23 kg/m^2^) and ALT (<40, ≥40 U/L). TC, TG, LDL-c, HDL-c, and non-HDL-c fell into quartiles, odds ratios (OR), the 95% confidence interval (CI) and trends for p which were used to assess the association between different lipid levels and hypertension. Moreover, age, sex, the BMI, FPG, ALT, AST, BUN, Scr, smoking status, alcohol consumption status, and family history of diabetes were adjusted in the multivariate logistic regression analysis, while statistical analyses were conducted by SPSS 26.0 and GraphPad 9.0.

The BN model is one of the probabilistic graphical models that combines probability theory and graph theory to reveal probabilistic dependencies among variables (nodes). It was constructed to further assess the relationship between baseline characteristics and incidence of hypertension, that is, to infer the probability of hypertension occurrence in the presence of multiple conditional variables. Here, it should be noted that the model was established based on the tree augmented native (TAN) algorithm in the model section of SPSS Modeler (version 18.0), and the parameter learning method was chosen as Bayesian adjustment of small cell counts (18). Arrows connecting two nodes represent that those two random variables are causally or unconditionally independent. If two nodes are not featured with arrows, the random variables are conditionally independent (19). The importance of the variables would be obtained. Based on the results of variable importance, the variables with the importance over 5% are selected for plotting the CPT using Netica 5.18 software.

## Results

Generally, the median age of the study population was 41 (IQR, 34–53). Baseline characteristics in the entire, non-hypertension and hypertension populations are presented in Table 1. In the hypertensive population, age, current drinker percentage, the BMI, FPG, ALT, AST and BUN were higher, while the percentage of current smokers and family history of diabetes was lower. In the lipid profile, TC, LDL-c, and non-HDL-c were higher in the hypertensive population.

**Table 1:** Baseline information of the overall males^*^.

	**Total (*n* = 62,957)**	**Non-hypertension (*n* = 51,222)**	**Hypertension (*n* = 11,735)**	***p*-value**
Age, years	41 (34–53)	39 (33–51)	52 (39–52)	<0.001
Current smoker (%)	6,657 (10.6)	5,503 (10.7)	1,154 (9.8)	0.004
Current drinker (%)	885 (1.4)	635 (1.2)	220 (1.9)	<0.001
Family history of diabetes (%)	1,041 (1.7)	879 (1.7)	162 (1.4)	0.011
SBP (mmHg)	122 (112–132)	118 (110–127)	144 (137–152)	<0.001
DBP (mmHg)	76 (69–84)	74 (68–80)	91 (85–96)	<0.001
BMI (kg/m^2^)	24.2 (22.2–26.3)	23.9 (21.9–25.9)	25.5 (23.5–27.7)	<0.001
FPG (mg/dl)	90 (82.8–97)	89.3 (82.44–95.8)	92.7 (85.3–100.8)	<0.001
ALT (U/L)	23 (16.9–34)	23 (16.5–33.4)	25 (18–37)	<0.001
AST (U/L)	23.6 (20–28.6)	23 (20–28)	25 (21–30)	<0.001
BUN (mmol/L)	4.81 (4.11–5.61)	4.8 (4.1–5.6)	4.89 (4.18–5.73)	<0.001
Scr (μmol/L)	79.9 (72.5–87.8)	77.9 (72.6–87.5)	77.8 (72–88.3)	0.336
**Lipid Profile**				
TC (mg/dl)	182.9 (162–206.4)	181.7 (160.1–204.9)	189.4 (167.4–212.6)	<0.001
TG (mg/dl)	97.5 (67.3–148)	97.5 (67.3–148)	97.5 (66.5–147.1)	0.274
LDL (mg/dl)	105.5 (89.7–123.3)	104.8 (88.9–122.2)	109 (92.8–126.8)	<0.001
HDL-c (mg/dl)	49.1 (42.1–56.4)	49.1 (42.1–56.44)	49.5 (42.1–56.8)	0.073
Non-HDL-c (mg/dl)	132.6 (112.1–156.2)	131 (111–154.6)	139.2 (118.1–162)	<0.001

* *Continuous data are expressed as median (interquartile range) due to the skewed distribution*.

*The p-value is a comparison between the normotension and hypertension groups*.

*FPG, fasting plasma glucose; TG, triglycerides; TC, total cholesterol; HDL-c, high-density lipoprotein cholesterol; LDL, low-density lipoprotein cholesterol; Scr, serum creatinine; BUN, blood urea nitrogen; ALT, alanine aminotransferase; AST, aspartate aminotransferase; BMI, Body mass index*.

### Correlation Between SBP, DBP, and Lipid Profiles

Spearman correlation analysis showed that SBP was positively correlated with TC [Spearman correlation coefficient (rho) rho = 0.1, *p* < 0.001], LDL-c (rho = 0.08, *p* < 0.001), HDL-c (rho = 0.01, *p* = 0.004), non-HDL-c (rho = 0.1, *p* <0.001), but not significantly correlated with TG (*p* = 0.51). DBP was positively correlated with TC (rho = 0.14, *p* < 0.001), LDL-c (rho = 0.11, *p* < 0.001) and non-HDL-c (rho = 0.15, *p* < 0.001), not significantly correlated with TG (*p* = 0.91), but negatively correlated with HDL-c (rho = −0.02, *p* < 0.001).

### Differences in Lipid Profiles Between Hypertensive and Non-hypertensive Groups According to Age, BMI, and ALT

Subgroup analyses were performed according to age, the BMI, and ALT, as shown in Table 2. In both the <60 and ≥60 years groups, TC, LDL-c, and non-HDL-c levels were higher in the hypertensive population, whereas HDL-c and TG differed slightly between the hypertensive and non-hypertensive populations. In the BMI <23 or ≥23 kg/m^2^ groups, TC, LDL-c, and non-HDL-c levels were higher in the hypertensive population, whereas HDL-c levels were higher in the hypertensive population. Similar results were observed in the ALT ≥ 40 U/L group while HDL-c was not significantly different in the hypertensive and non-hypertensive populations with ALT < 40 U/L.

**Table 2:** Subgroup analysis of lipid profiles differences between non-hypertension and hypertension according to age, BMI, and ALT^*^.

	**Non-hypertension**	**Hypertension**	***p*-value**	**Non-hypertension**	**Hypertension**	***p*-value**
Age	<60 years			≥60 years		
TC (mg/dl)	180.93 (159.30–203.35)	189.43 (166.23–212.63)	<0.001	188.66 (166.24–212.63)	192.14 (169.72–215.92)	<0.001
TG (mg/dl)	97.46 (67.34–147.96)	97.46 (66.45–146.19)	0.211	97.46 (67.34–147.30)	97.46 (66.45–147.08)	0.864
LDL (mg/dl)	104 (88.53–121.39)	108.25 (92.01–126.03)	<0.001	109.41 (92.78–127.2)	110.57 (94.72–128.93)	0.005
HDL-c (mg/dl)	49.1 (42.14–56.44)	49.1 (42.14–56.83)	0.242	49.1 (42.14–57.22)	49.48 (42.53–57.22)	0.741
Non-HDL-c (mg/dl)	130.28 (110.18–153.77)	138.4 (117.14–161.6)	<0.001	138.02 (116.37–161.6)	140.72 (120.23–163.92)	<0.001
BMI	<23 kg/m^2^			≥23 kg/m^2^		
TC (mg/dl)	173.97 (154.64–196.39)	181.9 (162.27–204.9)	<0.001	185.95 (164.69–209.15)	191.75 (169.72–214.56)	<0.001
TG (mg/dl)	97.46 (67.34–147.96)	97.46 (66.45–148.95)	0.967	97.46 (67.34–147.96)	97.46 (66.45–146.19)	0.221
LDL (mg/dl)	99.74 (85.05–116.37)	104.77 (88.90–122.17)	<0.001	107.86 (91.62–125.26)	110.18 (93.94–127.96)	<0.001
HDL-c (mg/dl)	51.42 (44.85–59.15)	52.58 (45.23–60.31)	<0.001	47.55 (40.98–54.9)	48.33 (41.75–56.06)	<0.001
Non-HDL-c (mg/dl)	121.0 (103.22–142.66)	128.74 (109.02–151.55)	<0.001	137.24 (116.37–160.05)	141.5 (121.0–164.69)	<0.001
ALT	<40 U/L			≥40 U/L		
TC (mg/dl)	179.77 (158.51–201.81)	188.27 (166.24–211.08)	<0.001	192.14 (169.81–216.11)	196.39 (173.97–220.36)	<0.001
TG (mg/dl)	97.46 (67.34–148.18)	97.46 (66.45–147.08)	0.123	97.46 (67.34–145.30)	97.46 (70.0–145.30)	0.2573
LDL (mg/dl)	103.61 (88.14–120.62)	108.25 (92.01–125.65)	<0.001	110.95 (94.33–129.51)	112.89 (95.49–131.83)	0.001
HDL-c (mg/dl)	49.48 (42.53–56.83)	49.48 (42.91–57.22)	0.09	46.78 (40.21–54.12)	47.55 (40.59–55.28)	0.006
Non-HDL-c (mg/dl)	128.74 (109.02–151.55)	137.24 (116.37–160.05)	<0.001	143.82 (121.78–168.17)	146.71 (126.03–171.17)	<0.001

* *Continuous data are expressed as median (interquartile range) due to the skewed distribution*.

*TG, triglycerides; TC, total cholesterol; HDL-c, high-density lipoprotein cholesterol; LDL, low-density lipoprotein cholesterol; ALT, alanine aminotransferase; AST, aspartate aminotransferase; BMI, Body mass index*.

### Multivariate Logistic Regression Analysis of Lipid Profiles and Hypertension Risk

Lipid profiles were classified into quartiles, with the first quartile (Q1) as a reference, as shown in Table 3. In the crude model, there was no significant association between TG and risk of hypertension, and still no association between TG and risk of hypertension was observed after full adjustment for multiple variables. TC, LDL-c, and non-HDL-c were positively related to the risk of hypertension (*p* for trend < 0.05). In the fully multivariate model, for every 1 mg/dl increase in TC, LDL-c and non-HDL, the risk of hypertension increased by 0.2% [1.002 (1.001–1.003)], 0.1% [1.001 (1.000–1.002)], and 0.1% [1.001 (1.000–1.002)], while in the Crude model and Model 1, HDL-c was not connected with the risk of hypertension (*p* for trend > 0.05), whereas after adjusting for the BMI, HDL-c became positively associated with the risk of hypertension (*p* for trend < 0.001). However, with 1 mg/dl increment in HDL-c, the risk of hypertension increased by 0.2% [1.002 (1.000–1.002)] after full adjustment for multiple variables.

**Table 3:** Multivariable logistic regression model evaluating the association between lipid levels and hypertension incidence.

	**Crude Model**		**Model 1**		**Model 2**		**Model 3**	
	**OR (95% CI)**	***p*-value**	**OR (95% CI)**	***p*-value**	**OR (95% CI)**	***p*-value**	**OR (95% CI)**	***p*-value**
**TG**								
Q 1 (≤67.34 mg/dl)	Ref		Ref		Ref		Ref	
Q 2 (67.34–97.46 mg/dl)	0.988 (0.933–1.046)	0.668	0.985 (0.928–1.045)	0.622	0.983 (0.925–1.044)	0.577	0.985 (0.926–1.048)	0.637
Q 3 (97.46–147.96 mg/dl)	0.988 (0.934–1.044)	0.662	0.987 (0.931–1.046)	0.651	0.983 (0.926–1.042)	0.559	0.989 (0.931–1.050)	0.711
Q 4 (≥147.96 mg/dl)	0.974 (0.921–1.031)	0.364	0.974 (0.918–1.032)	0.372	0.978 (0.921–1.038)	0.457	0.987 (0.929–1.049)	0.680
Per 1 mg/dl increase	1.000 (1.000–1.000)	0.407	1.000 (1.000–1.000)	0.416	1.000 (1.000–1.000)	0.691	1.000 (1.000–1.000)	0.905
*p* for trend	0.39		0.406		0.474		0.717	
**TC**								
Q 1 (≤161.98 mg/dl)	Ref		Ref		Ref		Ref	
Q 2 (161.98–182.86 mg/dl)	1.291 (1.215–1.372)	<0.001	1.188 (1.115–1.265)	<0.001	1.10 (1.031–1.173)	0.004	1.097 (1.027–1.172)	0.006
Q 3 (182.86–206.44 mg/dl)	1.510 (1.422–1.602)	<0.001	1.305 (1.227–1.388)	<0.001	1.150 (1.079–1.225)	<0.001	1.134 (1.063–1.209)	<0.001
Q 4 (≥206.44 mg/dl)	1.838 (1.734–1.948)	<0.001	1.438 (1.353–1.528)	<0.001	1.195 (1.123–1.272)	<0.001	1.159 (1.088–1.235)	<0.001
Per 1 mg/dl increase	1.007 (1.006–1.008)	<0.001	1.004 (1.003–1.005)	<0.001	1.002 (1.001–1.003)	<0.001	1.002 (1.001–1.003)	<0.001
*p* for trend	<0.001		<0.001		<0.001		<0.001	
**LDL**								
Q 1 (≤89.69 mg/dl)	Ref		Ref		Ref		Ref	
Q 2 (89.69–105.54 mg/dl)	1.158 (1.091–1.230)	<0.001	1.078 (1.013–1.147)	0.018	1.014 (0.852–1.081)	0.660	1.007 (0.944–1.074)	0.830
Q 3 (105.54–123.33 mg/dl)	1.340 (1.265–1.421)	<0.001	1.185 (1.116–1.259)	<0.001	1.069 (1.005–1.137)	0.035	1.06 (0.995–1.129)	0.07
Q 4 (≥123.33 mg/dl)	1.540 (1.453–1.631)	<0.001	1.250 (1.177–1.327)	<0.001	1.074 (1.010–1.142)	0.024	1.051 (0.987–1.119)	0.121
Per 1 mg/dl increase	1.006 (1.005–1.007)	<0.001	1.003 (1.002–1.004)	<0.001	1.001 (1.000–1.002)	0.007	1.001 (1.000–1.002)	0.057
*p* for trend	<0.001		<0.001		0.007		0.049	
**HDL-c**								
Q 1 (≤42.14 mg/dl)	Ref		Ref		Ref		Ref	
Q 2 (42.14–49.1 mg/dl)	0.994 (0.939–1.052)	0.833	1.016 (0.958–1.077)	0.599	1.114 (1.074–1.212)	<0.001	1.097 (1.027–1.172)	<0.001
Q 3 (49.1–56.44 mg/dl)	0.984 (0.929–1.043)	0.587	1.023 (0.964–1.086)	0.455	1.230 (1.156–1.308)	<0.001	1.134 (1.063–1.209)	<0.001
Q 4 (≥56.44 mg/dl)	1.057 (0.999–1.117)	0.053	1.043 (0.985–1.106)	0.151	1.359 (1.279–1.443)	<0.001	1.159 (1.088–1.235)	<0.001
Per 1 mg/dl increase	1.002 (1.001–1.004)	0.032	1.001 (0.999–1.003)	0.331	1.011 (1.009–1.013)	<0.001	1.002 (1.001–1.003)	<0.001
*p* for trend	0.075		0.15		<0.001		<0.001	
**Non-HDL-c**								
Q 1 (≤112.11 mg/dl)	Ref		Ref		Ref		Ref	
Q 2 (112.11–132.6 mg/dl)	1.288 (1.211–1.370)	<0.001	1.174 (1.102–1.252)	<0.001	1.047 (0.981–1.118)	0.165	1.044 (0.977–1.115)	0.202
Q 3 (132.6–156.19 mg/dl)	1.548 (1.459–1.643)	<0.001	1.314 (1.235–1.398)	<0.001	1.082 (1.016–1.153)	0.15	1.069 (1.002–1.140)	0.44
Q 4 (≥156.19 mg/dl)	1.853 (1.748–1.965)	<0.001	1.457 (1.371–1.549)	<0.001	1.105 (1.037–1.177)	0.002	1.080 (1.012–1.152)	0.020
Per 1 mg/dl increase	1.007 (1.006–1.008)	<0.001	1.004 (1.003–1.005)	<0.001	1.001 (1.000–1.002)	<0.001	1.001 (1.000–1.002)	0.010
*p* for trend	<0.001		<0.001		0.001		0.018	

*OR, odds ratio; CI, confidence interval; Q, quartile. Model 1 adjust age. Model 2 adjust age and BMI. Model 3 adjust Model 2+ FPG (mmol/L), ALT(U/L), BUN, Scr, smoking status (current smoker or not), drinking status (current drinker or not), family history of diabetes (Yes or No)*.

### Differences in Lipid Profiles at Different Blood Pressure Levels

**Figure 2:**
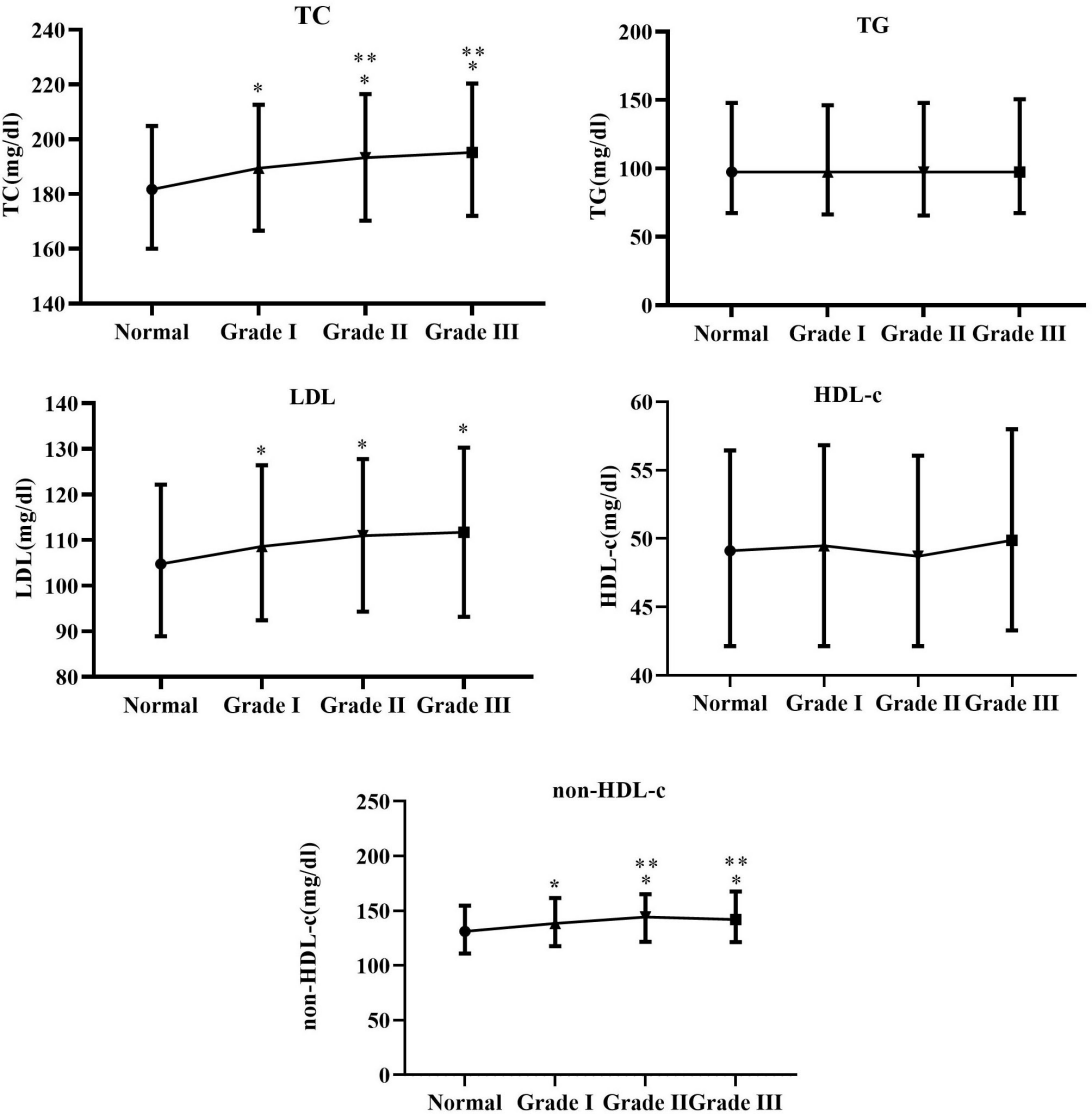
Between-group differences in TC, TG, LDL-c, HDL-c, and non-HDL at different blood pressure levels. *Indicates Grade I, Grade II and Grade III compared with normal group with *p*-value <0.05. **Indicates Grade II and Grade III compared with Grade I with *p*-value < 0.05.

As shown in Figure 2, TC, LDL-c, and non-HDL-c dramatically increased in the Grade I, Grade II, and Grade III groups (*p* <0.05), while TC and non-HDL-c levels were significantly higher in the Grade II and Grade III groups than those in the Grade I group (*p* < 0.05). TG and HDL-c were not obviously different in the normal, Grade I, Grade II and Grade III groups (*p* > 0.05).

### BN Model and CPT

**Figure 3:**
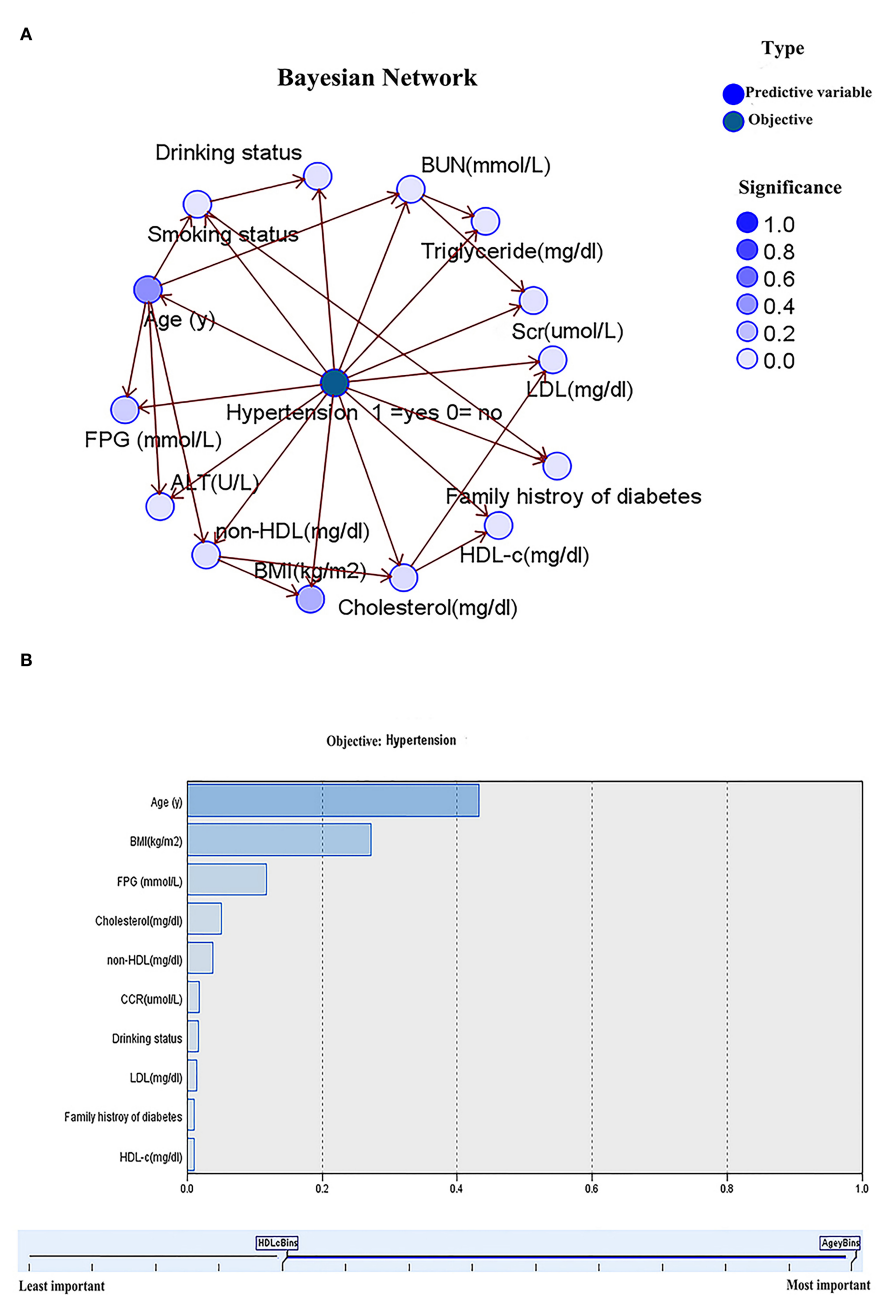
Bayesian network model for hypertension based on TAN algorithm **(A)**; Importance of predictors **(B)**.

From Figure 3A, age and the body mass index indirectly affect hypertension, while age alone directly influences smoking status, FPG, ALT, and non-HDL. In the lipid profiles, non-HDL directly affects TC generating a direct influence on LDL-c and HDL, indirectly affecting hypertension. Figure 3B shows the importance of baseline characteristics to the effect of hypertension incidence, when the importance of age, the BMI, FPG, and TC was 43.3, 27.2, 11.8, and 5.1%, respectively. Finally, conditional probability analysis on predictors with the importance over 5% was performed. As displayed in Table 4 and Supplementary Figure 1, the probability of not developing hypertension was the highest (92.6%) when age <60 years, the BMI <23 kg/m^2^, TC <240 mg/dl, and FPG <6.1 mmol/L, while that of developing hypertension reached the highest (50.3%) when age ≥60 years, the BMI ≥ 23 kg/m^2^, TC < 240 mg/dl, and FPG ≥ 6.1 mmol/L.

**Table 4:** Conditional probability table of hypertension.

**Age**	**BMI**	**TC**	**FPG**	**Hypertension (no)**	**Hypertension (yes)**
<60 years	<23 kg/m^2^	<240 mg/dl	<6.1 mmol/L	92.6%	7.4%
<60 years	<23 kg/m^2^	≥240 mg/dl	<6.1 mmol/L	89.2%	10.8%
<60 years	<23 kg/m^2^	<240 mg/dl	≥ 6.1 mmol/L	78%	22%
<60 years	<23 kg/m^2^	≥240 mg/dl	≥6.1 mmol/L	72.7%	27.3%
≥60 years	<23 kg/m^2^	<240 mg/dl	<6.1 mmol/L	70.3%	29.7%
≥60 years	<23 kg/m^2^	≥240 mg/dl	<6.1 mmol/L	68%	32%
≥60 years	<23 kg/m^2^	<240 mg/dl	≥6.1 mmol/L	55.8%	44.2%
≥60 years	<23 kg/m^2^	≥240 mg/dl	≥6.1 mmol/L	72.2%	27.8%
<60 years	≥23 kg/m^2^	<240 mg/dl	<6.1 mmol/L	82%	18%
<60 years	≥23 kg/m^2^	≥240 mg/dl	<6.1 mmol/L	75.9%	24.1%
<60 years	≥23 kg/m^2^	<240 mg/dl	≥6.1 mmol/L	67.5%	32.5%
<60 years	≥23 kg/m^2^	≥240 mg/dl	≥6.1 mmol/L	65%	35%
≥60 years	≥23 kg/m^2^	<240 mg/dl	<6.1 mmol/L	60.4%	39.6%
≥60 years	≥23 kg/m^2^	≥240 mg/dl	<6.1 mmol/L	55.9%	44.1%
≥60 years	≥23 kg/m^2^	<240 mg/dl	≥6.1 mmol/L	49.7%	50.3%
≥60 years	≥23 kg/m^2^	≥240 mg/dl	≥6.1 mmol/L	53.3%	46.7%

## Discussion

Our study was conducted on adult Chinese males. The results can be summarized as follows (1) Higher levels of TC, LDL-c and non-HDL were strongly associated with the incidence of hypertension, while HDL-c and TG were not significantly related to the incidence of hypertension. (2) SBP and DBP were positively associated with TC, LDL-c, and non-HDL levels and not significantly associated with TG. There existed a positive relationship between SBP and HDL, while DBP was negatively correlated with HDL. (3) After grouping the population by specific clinical characteristics (BMI and ALT), HDL-c was positively associated with the incidence of hypertension. (4) Multivariate logistic regression showed that TC, LDL-c, and non-HDL-c were positively associated with the incidence of hypertension, with no significant association with TG, while HDL-c shifted from no association to a positive association after adjustment for the BMI. At different blood pressure levels, TC and non-HDL-c levels were higher in the higher blood pressure group. (5) From Bayesian network analysis, age, the body mass index, FPG and TC were closely related to the onset of hypertension.

The incidence of hypertension is influenced by genetic and environmental factors, and its harmful effects are not only caused by the hemodynamic burden, but also due to many cardiovascular risk factors, such as metabolic syndrome (MetS), usually clustering in hypertensive patients (20). The metabolic syndrome, which includes visceral obesity, dyslipidemia, hyperglycemia, and hypertension, has become one of the major public health challenges worldwide (21). Our study shows that the BMI, dyslipidemia and impaired FBP are strongly associated with the onset of hypertension, and these factors are precisely the same as the metabolic syndrome spectrum. These factors may interact with the development of hypertension and share similar pathophysiological mechanisms. Some lifestyle changes such as lack of adequate physical activity, poor diet, high alcohol consumption, smoking and substance abuse provide favorable conditions for MetS and are also major risk factors for hypertension (22). Additionally, dyslipidemia and hypertension undergo similar pathophysiological mechanisms, such as endothelial cell damage, inflammation, oxidative stress, and atherosclerosis of the arteries (13, 23–25).

Several previous studies have examined the association between dyslipidemia and hypertension in non-Asian populations, and have shown that dyslipidemia is closely related to the development of hypertension (9–11, 26). However, the association of dyslipidemia with hypertension remains inconsistent in different populations and different patterns. The study by Otsuka et al. based on 14,215 Japanese working-aged males revealed that elevated TC, LDL-c, and non-HDL-c were associated with an increased risk of hypertension (13). However, He et al. observed that higher TG and lower HDL-C were connected with the incidence of hypertension, due to a study based on 9540 Chinese community residents aged >40 years (14). In line with a recent Chinese study (27), a positive association between HDL-c and risk of hypertension after stratifying for the BMI was observed, and the same association was also found in a multivariate logistic regression, which may be contrary to the current recognition of HDL as “good cholesterol”. The HDL-c function was independent of HDL-c levels, while it has been demonstrated that passive elevation of HDL-c levels does not add additional cardiovascular benefit (28–30), which may partially explain the result. It is interesting to note that a positive correlation between SBP and HDL-c and a negative correlation between DBP and HDL-c were found. The exact mechanism behind this finding remains unclear and may be related to the fact that HDL-C increases the bioavailability of nitric oxide in endothelial cells, which leads to vasodilation (31, 32), increases the peripheral vascular volume which can an increase in SBP and decreases vascular resistance lowering DBP.

Our study has the following advantages. Firstly, our cross-sectional study included a multicenter large sample population, whereas most previous studies were single-centered or had fewer cases. Secondly, differences in lipid profiles were analyzed for the entire, subgroups and populations with different blood pressure levels, while correlation and logistic analyses further clarified the association between lipid patterns and incidence of hypertension. These findings complement the current knowledge on the dyslipidemia and incidence of hypertension in Asian populations. Thirdly, the BN model revealed complex network relationships between hypertension and its associated factors, providing some new insights into the relationship between lipids and hypertension in analyzing disease-related factors.

Inevitably, our study is featured with the following limitations. (1) Firstly, the type of our study was cross-sectional study, which could not present a causal relationship between dyslipidemia and hypertension. (2) Secondly, our study population was adult males, recruited from 11 affluent cities in China, and was not so representative. (3) Thirdly, baseline information on patients' history of chronic disease and medication use was not available, and its effect on the dyslipidemia and hypertension incidence remains unclear.

## Conclusion

Elevated TC, LDL-c, and non-HDL-c were associated with incidence of hypertension in Chinese adult males, whereas TG was not significantly related. The relations between HDL-c and hypertension incidence shifted from no association to a positive correlation after adjusting for the BMI. The BN model showed that age, the BMI, FPG, and TC were closely connected with hypertension incidence.

## Data Availability Statement

The datasets presented in this study can be found in online repositories. The names of the repository/repositories and accession number(s) can be found in the article/Supplementary Material.

## Ethics Statement

The studies involving human participants were reviewed and approved by Rich Healthcare Group Review Board. Written informed consent for participation was not required for this study in accordance with the national legislation and the institutional requirements.

## Author Contributions

WKC designed this topic. WKC and SWC drafted, analyzed, interpreted this study, and critically reviewed the study. Both authors finally agreed to submit the manuscript.

## Funding

WKC was funded by China Scholarship Council (CSC No. 202009370095).

## Conflict of Interest

The authors declare that the research was conducted in the absence of any commercial or financial relationships that could be construed as a potential conflict of interest.

## Publisher's Note

All claims expressed in this article are solely those of the authors and do not necessarily represent those of their affiliated organizations, or those of the publisher, the editors and the reviewers. Any product that may be evaluated in this article, or claim that may be made by its manufacturer, is not guaranteed or endorsed by the publisher.

## Supplementary Material

Supplementary Figure 1 The diagnosis result for hypertension by Netica.
