# Supplementary material for: Relationship Between Lipid Profiles and Hypertension: A Cross-Sectional Study of 62,957 Chinese Adult Males
Source: Front Public Health. 2022 May 18;10:895499. doi: 10.3389/fpubh.2022.895499 (PMC9159857; doi:10.3389/fpubh.2022.895499)
Supplement: Supplementary file 1 [file Table_1.DOCX]

| ***Table 1*. Baseline information of the overall males*** | | | | |
| --- | --- | --- | --- | --- |
|  | Total  (n=62,957) | Non-Hypertension  (n=51,222) | Hypertension  (n=11,735) | p value |
| Age, years | 41(34-53) | 39(33-51) | 52(39-52) | ＜0.001 |
| Current smoker (%) | 6657(10.6) | 5503(10.7) | 1154(9.8) | 0.004 |
| Current drinker (%) | 885(1.4) | 635(1.2) | 220(1.9) | ＜0.001 |
| Family history of diabetes (%) | 1041(1.7) | 879(1.7) | 162(1.4) | 0.011 |
| SBP (mmHg) | 122(112-132) | 118(110-127) | 144(137-152) | ＜0.001 |
| DBP (mmHg) | 76(69-84) | 74(68-80) | 91(85-96) | ＜0.001 |
| BMI (kg/m^2^) | 24.2(22.2-26.3) | 23.9(21.9-25.9) | 25.5(23.5-27.7) | ＜0.001 |
| FPG (mg/dl) | 90(82.8-97) | 89.3(82.44-95.8) | 92.7(85.3-100.8) | ＜0.001 |
| ALT (U/L) | 23(16.9-34) | 23(16.5-33.4) | 25(18-37) | ＜0.001 |
| AST (U/L) | 23.6(20-28.6) | 23(20-28) | 25(21-30) | ＜0.001 |
| BUN (mmol/L) | 4.81(4.11-5.61) | 4.8(4.1-5.6) | 4.89(4.18-5.73) | ＜0.001 |
| Scr (μmol/L) | 79.9(72.5-87.8) | 77.9(72.6-87.5) | 77.8(72-88.3) | 0.336 |
| **Lipid Profile** |  |  |  |  |
| TC (mg/dl) | 182.9(162-206.4) | 181.7(160.1-204.9) | 189.4(167.4-212.6) | ＜0.001 |
| TG (mg/dl) | 97.5(67.3-148) | 97.5(67.3-148) | 97.5(66.5-147.1) | 0.274 |
| LDL (mg/dl) | 105.5(89.7-123.3) | 104.8(88.9-122.2) | 109(92.8-126.8) | ＜0.001 |
| HDL-c (mg/dl) | 49.1 (42.1-56.4) | 49.1(42.1-56.44) | 49.5(42.1-56.8) | 0.073 |
| Non-HDL-c (mg/dl) | 132.6(112.1-156.2) | 131(111-154.6) | 139.2(118.1-162) | ＜0.001 |

*Continuous data are expressed as median (interquartile range) due to the skewed distribution.

The p-value is a comparison between the normotension and hypertension groups.

FPG, fasting plasma glucose. TG, triglycerides. TC, total cholesterol. HDL-c, high-density lipoprotein cholesterol. LDL, low-density lipoprotein cholesterol. Scr, serum creatinine. BUN, blood urea nitrogen. ALT, alanine aminotransferase. AST, aspartate aminotransferase. BMI, Body mass index.
