# Supplementary material for: Relationship Between Lipid Profiles and Hypertension: A Cross-Sectional Study of 62,957 Chinese Adult Males
Source: Front Public Health. 2022 May 18;10:895499. doi: 10.3389/fpubh.2022.895499 (PMC9159857; doi:10.3389/fpubh.2022.895499)
Supplement: Supplementary file 2 [file Table_2.DOCX]

| **Table 2. Subgroup analysis of lipid profiles differences between non-hypertension and hypertension according to** **age, BMI, and ALT*.** | | | | | | |
| --- | --- | --- | --- | --- | --- | --- |
|  | Non-hypertension | Hypertension | p-value | Non-hypertension | Hypertension | p-value |
| **Age** | ＜60 years | |  | ≥60 years | |  |
| TC (mg/dl) | 180.93(159.30-203.35) | 189.43(166.23-212.63) | ＜0.001 | 188.66(166.24-212.63) | 192.14(169.72-215.92) | ＜0.001 |
| TG (mg/dl) | 97.46(67.34-147.96) | 97.46(66.45-146.19) | 0.211 | 97.46(67.34-147.30) | 97.46(66.45-147.08) | 0.864 |
| LDL (mg/dl) | 104(88.53-121.39) | 108.25(92.01-126.03) | ＜0.001 | 109.41(92.78-127.2) | 110.57(94.72-128.93) | 0.005 |
| HDL-c (mg/dl) | 49.1(42.14-56.44) | 49.1(42.14-56.83) | 0.242 | 49.1(42.14-57.22) | 49.48(42.53-57.22) | 0.741 |
| Non-HDL-c (mg/dl) | 130.28(110.18-153.77) | 138.4(117.14-161.6) | ＜0.001 | 138.02(116.37-161.6) | 140.72(120.23-163.92) | ＜0.001 |
| **BMI** | ＜23 kg/m^2^ | |  | ≥23 kg/m^2^ | |  |
| TC (mg/dl) | 173.97(154.64-196.39) | 181.9(162.27-204.9) | ＜0.001 | 185.95(164.69-209.15) | 191.75(169.72-214.56) | ＜0.001 |
| TG (mg/dl) | 97.46(67.34-147.96) | 97.46(66.45-148.95) | 0.967 | 97.46(67.34-147.96) | 97.46(66.45-146.19) | 0.221 |
| LDL (mg/dl) | 99.74(85.05-116.37) | 104.77(88.90-122.17) | ＜0.001 | 107.86(91.62-125.26) | 110.18(93.94-127.96) | ＜0.001 |
| HDL-c (mg/dl) | 51.42(44.85-59.15) | 52.58(45.23-60.31) | ＜0.001 | 47.55(40.98-54.9) | 48.33(41.75-56.06) | ＜0.001 |
| Non-HDL-c (mg/dl) | 121.0(103.22-142.66) | 128.74(109.02-151.55) | ＜0.001 | 137.24(116.37-160.05) | 141.5(121.0-164.69) | ＜0.001 |
| **ALT** | ＜40 U/L | |  | ≥40 U/L | |  |
| TC (mg/dl) | 179.77(158.51-201.81) | 188.27(166.24-211.08) | ＜0.001 | 192.14(169.81-216.11) | 196.39(173.97-220.36) | ＜0.001 |
| TG (mg/dl) | 97.46(67.34-148.18) | 97.46(66.45-147.08) | 0.123 | 97.46(67.34-145.30) | 97.46(70.0-145.30) | 0.2573 |
| LDL (mg/dl) | 103.61(88.14-120.62) | 108.25(92.01-125.65) | ＜0.001 | 110.95(94.33-129.51) | 112.89(95.49-131.83) | 0.001 |
| HDL-c (mg/dl) | 49.48(42.53-56.83) | 49.48(42.91-57.22) | 0.09 | 46.78(40.21-54.12) | 47.55(40.59-55.28) | 0.006 |
| Non-HDL-c (mg/dl) | 128.74(109.02-151.55) | 137.24(116.37-160.05) | ＜0.001 | 143.82(121.78-168.17) | 146.71(126.03-171.17) | ＜0.001 |

*Continuous data are expressed as median (interquartile range) due to the skewed distribution.

TG, triglycerides. TC, total cholesterol. HDL-c, high-density lipoprotein cholesterol. LDL, low-density lipoprotein cholesterol. ALT, alanine aminotransferase. AST, aspartate aminotransferase. BMI, Body mass index.
