# Supplementary material for: Relationship Between Lipid Profiles and Hypertension: A Cross-Sectional Study of 62,957 Chinese Adult Males
Source: Front Public Health. 2022 May 18;10:895499. doi: 10.3389/fpubh.2022.895499 (PMC9159857; doi:10.3389/fpubh.2022.895499)
Supplement: Supplementary file 3 [file Table_3.DOCX]

| **Table 3. Multivariable logistic regression model evaluating the association between lipid levels and hypertension incidence.** | | | | | | | | |
| --- | --- | --- | --- | --- | --- | --- | --- | --- |
|  | Crude Model | | Model 1 | | Model 2 | | Model 3 | |
| **TG** | OR (95% CI) | *p*-value | OR (95% CI) | *p*-value | OR (95% CI) | *p*-value | OR (95% CI) | *p*-value |
| Q 1 (≤67.34 mg/dl) | Ref |  | Ref |  | Ref |  | Ref |  |
| Q 2(67.34-97.46 mg/dl) | 0.988(0.933-1.046) | 0.668 | 0.985(0.928-1.045) | 0.622 | 0.983(0.925-1.044) | 0.577 | 0.985(0.926-1.048) | 0.637 |
| Q 3(97.46-147.96 mg/dl) | 0.988(0.934-1.044) | 0.662 | 0.987(0.931-1.046) | 0.651 | 0.983(0.926-1.042) | 0.559 | 0.989(0.931-1.050) | 0.711 |
| Q 4(≥147.96 mg/dl) | 0.974(0.921-1.031) | 0.364 | 0.974(0.918-1.032) | 0.372 | 0.978(0.921-1.038) | 0.457 | 0.987(0.929-1.049) | 0.680 |
| Per 1 mg/dl increase | 1.000(1.000-1.000) | 0.407 | 1.000(1.000-1.000) | 0.416 | 1.000(1.000-1.000) | 0.691 | 1.000(1.000-1.000) | 0.905 |
| *p* for trend | 0.39 |  | 0.406 |  | 0.474 |  | 0.717 |  |
| **TC** |  |  |  |  |  |  |  |  |
| Q 1 (≤161.98 mg/dl) | Ref |  | Ref |  | Ref |  | Ref |  |
| Q 2(161.98-182.86 mg/dl) | 1.291(1.215-1.372) | ＜0.001 | 1.188(1.115-1.265) | ＜0.001 | 1.10(1.031-1.173) | 0.004 | 1.097(1.027-1.172) | 0.006 |
| Q 3(182.86-206.44 mg/dl) | 1.510(1.422-1.602) | ＜0.001 | 1.305(1.227-1.388) | ＜0.001 | 1.150(1.079-1.225) | ＜0.001 | 1.134(1.063-1.209) | ＜0.001 |
| Q 4(≥206.44 mg/dl) | 1.838(1.734-1.948) | ＜0.001 | 1.438(1.353-1.528) | ＜0.001 | 1.195(1.123-1.272) | ＜0.001 | 1.159(1.088-1.235) | ＜0.001 |
| Per 1 mg/dl increase | 1.007(1.006-1.008) | ＜0.001 | 1.004(1.003-1.005) | ＜0.001 | 1.002(1.001-1.003) | ＜0.001 | 1.002(1.001-1.003) | ＜0.001 |
| *p* for trend | ＜0.001 |  | ＜0.001 |  | ＜0.001 |  | ＜0.001 |  |
| **LDL** |  |  |  |  |  |  |  |  |
| Q 1(≤89.69 mg/dl) | Ref |  | Ref |  | Ref |  | Ref |  |
| Q 2(89.69-105.54 mg/dl) | 1.158(1.091-1.230) | ＜0.001 | 1.078(1.013-1.147) | 0.018 | 1.014(0.852-1.081) | 0.660 | 1.007(0.944-1.074) | 0.830 |
| Q 3(105.54-123.33 mg/dl) | 1.340(1.265-1.421) | ＜0.001 | 1.185(1.116-1.259) | ＜0.001 | 1.069(1.005-1.137) | 0.035 | 1.06(0.995-1.129) | 0.07 |
| Q 4(≥123.33 mg/dl) | 1.540(1.453-1.631) | ＜0.001 | 1.250(1.177-1.327) | ＜0.001 | 1.074(1.010-1.142) | 0.024 | 1.051(0.987-1.119) | 0.121 |
| Per 1 mg/dl increase | 1.006(1.005-1.007) | ＜0.001 | 1.003(1.002-1.004) | ＜0.001 | 1.001(1.000-1.002) | 0.007 | 1.001(1.000-1.002) | 0.057 |
| *p* for trend | ＜0.001 |  | ＜0.001 |  | 0.007 |  | 0.049 |  |
| **HDL-c** |  |  |  |  |  |  |  |  |
| Q 1(≤42.14 mg/dl) | Ref |  | Ref |  | Ref |  | Ref |  |
| Q 2(42.14-49.1 mg/dl) | 0.994(0.939-1.052) | 0.833 | 1.016(0.958-1.077) | 0.599 | 1.114(1.074-1.212) | ＜0.001 | 1.097(1.027-1.172) | ＜0.001 |
| Q 3(49.1-56.44 mg/dl) | 0.984(0.929-1.043) | 0.587 | 1.023(0.964-1.086) | 0.455 | 1.230(1.156-1.308) | ＜0.001 | 1.134(1.063-1.209) | ＜0.001 |
| Q 4(≥56.44 mg/dl) | 1.057(0.999-1.117) | 0.053 | 1.043(0.985-1.106) | 0.151 | 1.359(1.279-1.443) | ＜0.001 | 1.159(1.088-1.235) | ＜0.001 |
| Per 1 mg/dl increase | 1.002(1.001-1.004) | 0.032 | 1.001(0.999-1.003) | 0.331 | 1.011(1.009-1.013) | ＜0.001 | 1.002(1.001-1.003) | ＜0.001 |
| *p* for trend | 0.075 |  | 0.15 |  | ＜0.001 |  | ＜0.001 |  |
| **non-HDL-c** |  |  |  |  |  |  |  |  |
| Q 1(≤112.11 mg/dl) | Ref |  | Ref |  | Ref |  | Ref |  |
| Q 2(112.11-132.6 mg/dl) | 1.288(1.211-1.370) | ＜0.001 | 1.174(1.102-1.252) | ＜0.001 | 1.047(0.981-1.118) | 0.165 | 1.044(0.977-1.115) | 0.202 |
| Q 3(132.6-156.19 mg/dl) | 1.548(1.459-1.643) | ＜0.001 | 1.314(1.235-1.398) | ＜0.001 | 1.082(1.016-1.153) | 0.15 | 1.069(1.002-1.140) | 0.44 |
| Q 4(≥156.19 mg/dl) | 1.853(1.748-1.965) | ＜0.001 | 1.457(1.371-1.549) | ＜0.001 | 1.105(1.037-1.177) | 0.002 | 1.080(1.012-1.152) | 0.020 |
| Per 1 mg/dl increase | 1.007(1.006-1.008) | ＜0.001 | 1.004(1.003-1.005) | ＜0.001 | 1.001(1.000-1.002) | ＜0.001 | 1.001(1.000-1.002) | 0.010 |
| *p* for trend | ＜0.001 |  | ＜0.001 |  | 0.001 |  | 0.018 |  |

OR, odds ratio. CI, confidence interval. Q, quartile. Model 1 adjust age. Model 2 adjust age and BMI. Model 3 adjust Model 2+ FPG (mmol/L), ALT(U/L), BUN, Scr, smoking status (current smoker or not), drinking status (current drinker or not), family history of diabetes (Yes or No).
