# Supplementary material for: Relationship Between Lipid Profiles and Hypertension: A Cross-Sectional Study of 62,957 Chinese Adult Males
Source: Front Public Health. 2022 May 18;10:895499. doi: 10.3389/fpubh.2022.895499 (PMC9159857; doi:10.3389/fpubh.2022.895499)
Supplement: Supplementary file 4 [file Table_4.DOCX]

| ***Table 4*. Conditional probability table of hypertension** | | | | | |
| --- | --- | --- | --- | --- | --- |
| **Age** | **BMI** | **TC** | **FPG** | **Hypertension**  **(No)** | **Hypertension**  **(Yes)** |
| ＜60 years | ＜23 kg/m^2^ | ＜240 mg/dl | ＜6.1 mmol/L | 92.6% | 7.4% |
| ＜60 years | ＜23 kg/m^2^ | ≥240 mg/dl | ＜6.1 mmol/L | 89.2% | 10.8% |
| ＜60 years | ＜23 kg/m^2^ | ＜240 mg/dl | ≥ 6.1 mmol/L | 78% | 22% |
| ＜60 years | ＜23 kg/m^2^ | ≥240 mg/dl | ≥6.1 mmol/L | 72.7% | 27.3% |
| ≥60 years | ＜23 kg/m^2^ | ＜240 mg/dl | ＜6.1 mmol/L | 70.3% | 29.7% |
| ≥60 years | ＜23 kg/m^2^ | ≥240 mg/dl | ＜6.1 mmol/L | 68% | 32% |
| ≥60 years | ＜23 kg/m^2^ | ＜240 mg/dl | ≥6.1 mmol/L | 55.8% | 44.2% |
| ≥60 years | ＜23 kg/m^2^ | ≥240 mg/dl | ≥6.1 mmol/L | 72.2% | 27.8% |
| ＜60 years | ≥23 kg/m^2^ | ＜240 mg/dl | ＜6.1 mmol/L | 82% | 18% |
| ＜60 years | ≥23 kg/m^2^ | ≥240 mg/dl | ＜6.1 mmol/L | 75.9% | 24.1% |
| ＜60 years | ≥23 kg/m^2^ | ＜240 mg/dl | ≥6.1 mmol/L | 67.5% | 32.5% |
| ＜60 years | ≥23 kg/m^2^ | ≥240 mg/dl | ≥6.1 mmol/L | 65% | 35% |
| ≥60 years | ≥23 kg/m^2^ | ＜240 mg/dl | ＜6.1 mmol/L | 60.4% | 39.6% |
| ≥60 years | ≥23 kg/m^2^ | ≥240 mg/dl | ＜6.1 mmol/L | 55.9% | 44.1% |
| ≥60 years | ≥23 kg/m^2^ | ＜240 mg/dl | ≥6.1 mmol/L | 49.7% | 50.3% |
| ≥60 years | ≥23 kg/m^2^ | ≥240 mg/dl | ≥6.1 mmol/L | 53.3% | 46.7% |
