# Supplementary figures and images for: Relationship Between Lipid Profiles and Hypertension: A Cross-Sectional Study of 62,957 Chinese Adult Males
Source: Front Public Health. 2022 May 18;10:895499. doi: 10.3389/fpubh.2022.895499 (PMC9159857; doi:10.3389/fpubh.2022.895499)

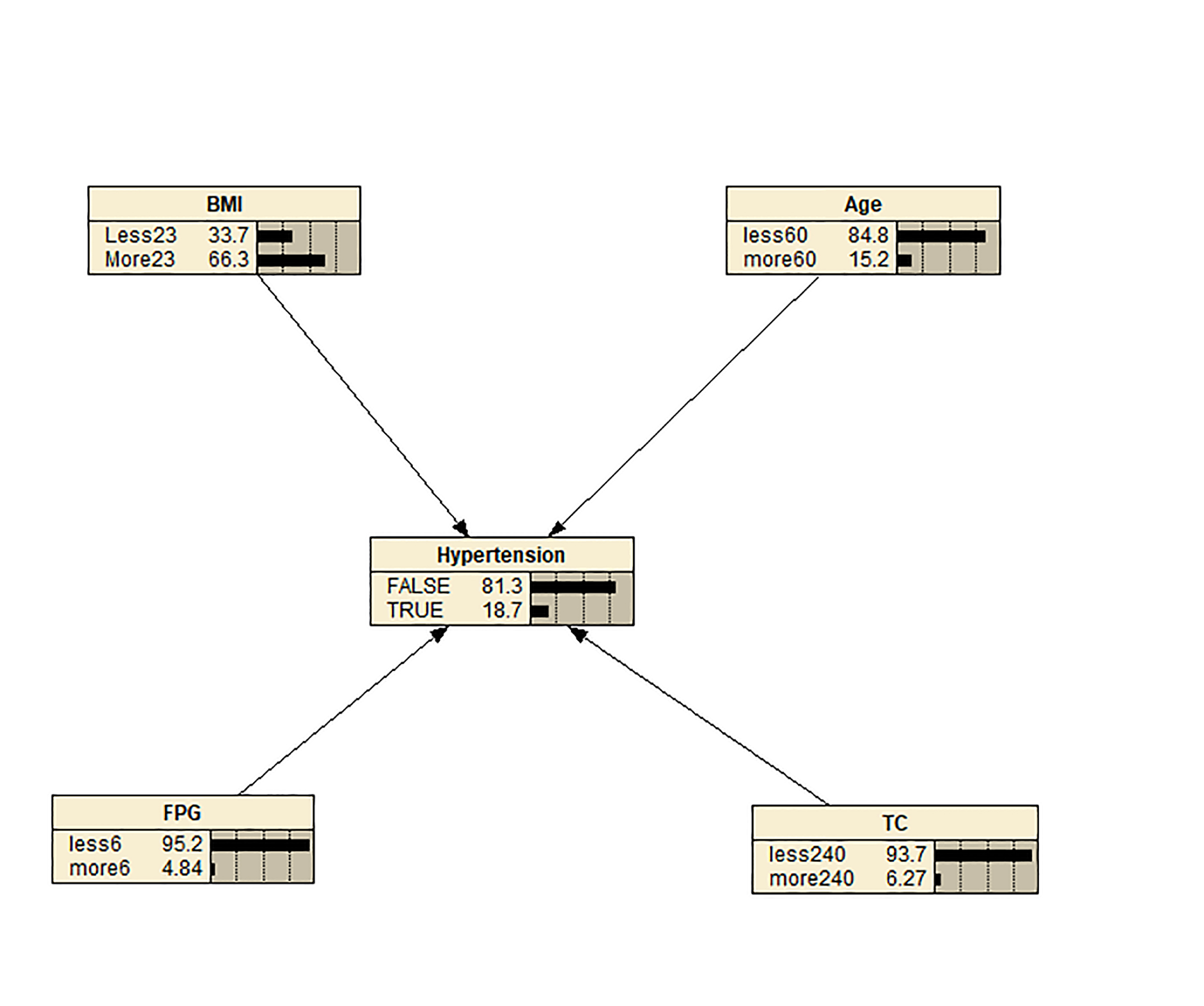

Supplement: Supplementary Figure 1 — The diagnosis result for hypertension by Netica. [file Image_1.JPEG]
